# Brain SPECT as an Imaging Biomarker for Evaluating Effects of Novel Treatments in Psychiatry–A Case Series

**DOI:** 10.3389/fpsyt.2021.713141

**Published:** 2022-01-13

**Authors:** Steven R. D. Best, Natalie Haustrup, Dan G. Pavel

**Affiliations:** ^1^The Neuroscience Center, Deerfield, IL, United States; ^2^Haustrup Scientific Consulting, Cork, Ireland; ^3^PathFinder Brain SPECT, Deerfield, IL, United States

**Keywords:** SPECT, biomarker, CTK, HBOT, depression, psychiatry, brain, treatment

## Abstract

The difficulties of evaluating patients with complex neuropsychiatric conditions and prescribing appropriate treatments are well known. Imaging complements clinical assessments and allows a clinician to narrow the differential diagnosis by facilitating accurate and efficient evaluation. This is particularly relevant to neuropsychiatric conditions that are often diagnosed using a trial-and error process of exclusion. Single Photon Emission Computed Tomography (SPECT) is a functional brain imaging procedure that allows practitioners to measure the functional changes of gray matter structures based on regional cerebral blood flow (rCBF). The accurate diagnosis and treatment selection in psychiatry is challenging due to complex cases and frequent comorbidities. However, such complex neuropsychiatric conditions are increasingly benefitting from new treatment approaches, in addition to established medications. Among these are combination transcranial magnetic stimulation with ketamine infusions (CTK), hyperbaric oxygen therapy (HBOT) and perispinal administration of etanercept (PSE). This article provides readers with six case study examples that demonstrate how brain SPECT imaging can be used, both as a diagnostic tool, and as a potential biomarker for monitoring and evaluating novel treatments for patients with complex neuropsychiatric conditions. Six patients were assessed in our clinic and baseline brain SPECT imagesTourettes and a long history of alcohol were visually compared with SPECT images collected after periods of treatment with CTK or HBOT followed by PSE. This retrospective review demonstrates the clinical utility of these novel treatments and describes how SPECT imaging can complement standard diagnostic assessments. A novel display technique for SPECT images is described and we argue that SPECT imaging can be used for monitoring biomarker for clinical change.

## Introduction

While experienced psychiatrists may be able to diagnose patients with neuropsychiatric conditions based on behavioral criteria, functional brain imaging tools can inform the clinician of their underlying neurobiology. Functional brain imaging can therefore provide clinicians with insightful information that enables them to narrow the differential diagnosis and to monitor and to evaluate any therapeutic benefit of the treatment (1). Single Photon Emission Computed Tomography (SPECT) is a functional brain imaging procedure that displays the functional status in the whole gray matter volume. A radiotracer is administered to the patient and is transported *via* the bloodstream and is quickly removed through normal kidney excretion. During circulation, some radiotracer is taken up by the brain tissue with the uptake of radiotracer dependent on the regional cerebral blood flow (rCBF). The detection of the radiotracer uptake across the brain allows the clinician to identify areas of both underperfusion (hypofunctioning) and of hyperperfusion (hyperfunctioning). Brain SPECT also has the functionality to detect the presence of comorbidity that can occur due to a variety of causes, including neurodevelopmental problems, traumatic brain injury, neuro-inflammation, non-convulsive epilepsy, neurotoxic exposure and nutritional deficiencies all of which contribute to altering the blood flow levels in various gray matter structures.

SPECT generates a three-dimensional (3-D) mapped representation of the brain that can be presented with color-coded intensities proportional to rCBF and correlating with the function in that region. Accurate and reliable visual interpretation of brain SPECT relies on optimizing the presentation of images using effective display tools and techniques, which are demonstrated herein. Diagnosis can also benefit from the complementary information exhibited by SPECT images displayed in a variety of formats including slices, surfaces and volumes. The optimal approach to accomplish accurate and efficient interpretation of brain imaging modalities is debated by researchers and clinicians in the literature (2–7), with discussions primarily centered on either conventional visual analysis by an experienced investigator and/or quantification techniques including voxel-wise analysis or region of interest (ROI) approaches (8–12). SPECT image quantification within clinical research typically identifies statistically significant differences based on mean group values, which do not always equate to individual differences, which is typically the focus in clinical practice (8). Therefore, the visual interpretation of individual SPECT images remains a foundational skill within clinical practice and quantification is considered favorable for larger studies for identifying trends (8, 13).

However, SPECT imaging is underutilized in clinical practice despite a growing, evidence-based foundation for its application in numerous indications relevant to psychiatric practice (14–23). This underutilization is particularly unfortunate as SPECT is an easy-to-perform, non-invasive procedure and remains among the least expensive neuroimaging tools available (24, 25). The historic underutilization of neuroimaging techniques in psychiatry has also led to the consequential inadequate biological understanding of neuropsychiatric conditions (26). Without such biological understanding it is also difficult to identify meaningful biomarkers for diagnosis, prognosis or risk, which is particularly relevant as psychiatric treatments can lead to biological changes (24, 26, 27). In addition, neuropsychiatric conditions are often diagnosed using a process of exclusion and additional information from brain SPECT imaging can complement the information gathered from clinical assessments. Given the current underutilization of SPECT, additional work is required to integrate the brain SPECT information in a more precise clinical context, given the extent of comorbidities present in many neuropsychiatric conditions.

Previous studies have demonstrated how SPECT can be utilized to diagnose psychiatric disorders (3, 28, 29) and to evaluate established treatments (4, 30–32). The accurate diagnosis and treatment selection is complicated in psychiatry by complex cases and frequent comorbidities (2). However, these conditions are increasingly benefitting from new treatment approaches, including Transcranial Magnetic Stimulation (TMS), combinations of TMS with ketamine infusion (CTK) and Hyperbaric Oxygen Therapy (HBOT) with perispinal administration of etanercept (PSE) (33–36)]. TMS is a non-invasive technique for stimulation of the brain that can induce antidepressant and anti-manic effects, however treatment response can be slow (37–39). Ketamine is effective in reducing depressive symptoms (40) *via* multiple mechanisms of action, including modulating signaling that stimulates neurogenesis and neuroplasticity, as well as acting as a tumor necrosis factor (TNF)-alpha inhibitor resulting in an anti-inflammatory effect (41–43). CTK is a patented procedure and studies have indicated that CTK is an effective, long-term therapy for patients with various neuropsychiatric conditions, whereby the coincident administration of ketamine allowed for higher TMS intensities than otherwise would be tolerated by patients (33, 44–46). Treatment with HBOT followed by PSE has also been identified as a possible treatment for cognitive impairment (35, 36, 47, 48). HBOT is believed to have anti-inflammatory effects by reducing excess pro-inflammatory cytokine activation, such as TNF-alpha, and facilitates improvement by provocation of stem cell activity, which can lessen the neurological impact of brain injuries (49–51). PSE injections modulate TNF-alpha directly to the central nervous system and act to normalize the inflammatory response in stroke, traumatic brain injury, and encephalopathic conditions (52). Despite supportive evidence of the clinical utility of novel treatments, such as CTK and HBOT with PSE, further investigation of these combination treatments is required.

This article builds on the current literature and presents a retrospective review of a case series including six patients with complex neuropsychiatric presentations. Baseline brain SPECT images were visually compared with SPECT images collected after periods of treatment with three novel treatments of CTK or HBOT followed by PSE during routine clinical practice. In this article we also describe a novel SPECT imaging display technique, present evidence of the clinical utility of CTK and HBOT with PSE and we propose that SPECT can be used as an imaging biomarker for monitoring and evaluating clinical change.

## Materials and Methods

### Study Cases

Six patients presented to our clinic with disabling neuropsychiatric conditions of various causes following extensive unsuccessful periods of treatments. The conditions and comorbidities differed for each patient (I-VI). Treatments were selected for each patient following the clinical assessment of the patient. The six cases presented herein were purposefully selected as they demonstrate the usefulness of brain SPECT imaging in evaluating patients with neuropsychiatric conditions and to monitor their response to treatment. An overview of the patients, their diagnosis and treatment are presented in Table 1 with more thorough details of patient histories, selected treatments and outcomes presented in the Results section. All subjects consented to the use of their data and information for the research purposes described herein.

**Table 1:** Characteristics of patients with complex neuropsychiatric conditions (*n* = *6*) with selected treatment.

**Patient**	**Age**	**Sex**	**Diagnosis**	**Treatment**
I	62	F	Treatment-resistant depression (TRD) as well as grief and the effects of prolonged polypharmacy	CTK
II	34	F	Regulatory disorder of childhood, post-head injury epilepsy, reflex sympathetic dystrophy (RSD)	HBOT and PSE
III	54	M	Childhood-onset Tourettes, long history of alcohol abuse, severe depression, fatigue and sleep apnea	CTK
IV	55	F	Major depressive disorder (MDD), panic/agoraphobia, chronic back pain, frequent headaches	CTK
V	77	M	Dementia with major cognitive deficits and aphasia	HBOT and PSE
VI	43	M	Bipolar II, lifelong symptoms of depression, anxiety, impulsive behavior and family stressors	CTK

### Novel Treatment Options

SPECT imaging was used to identify the extent and severity of hypoperfused areas, which complemented the standard clinical assessment data collected. Treatment decisions were based on the full baseline assessment and patients were either treated with CTK or HBOT followed by PSE. The CTK procedure has been described in detail previously (9). Patients who present at our clinic with TRD are treated with CTK before, or instead of, electroconvulsive therapy (ECT) or TMS or ketamine administered independently, based on evidence that CTK offers benefits over these established treatments for patients with TRD (33, 46). In brief, four patients treated with CTK received TMS (30 min) and 5 min after the commencement of TMS, intravenous infusions of the NMDA-receptor inhibitor, ketamine, began (20 min). The TMS (1 Hz) was applied continuously for 30 min at a power output setting equivalent to 130% of motor threshold (MT). A biomarker-dependent dosing strategy was applied, whereby ketamine was gradually titrated in small increments until the patient entered a mildly cataleptic state. Catalepsy refers to the neuromuscular condition characterized by muscular rigidity and fixity of posture regardless of external stimuli, as well as markedly decreased sensitivity to pain. Titrations began at 20 mg, with an average dosage range of 0.4–2.3 mg/kg (full range from 0.2 to 4.7 mg/kg). Once the patient began to stiffen or posture, the ketamine infusions could be discontinued. Following the completion of the ketamine infusion, the TMS would continue for a further 5 min, after which the CTK procedure was complete. Frequency of treatment is dependent on patient responsiveness (typically 10–30 sessions).

Two patients who presented to this clinic with treatment refractory illness in the context of traumatic brain injury (TBI) or mild TBI (mTBI) were treated with HBOT followed by PSE. Previous experiences of treating patients have indicated that the benefits of HBOT and PSE injection may be cumulative (53). HBOT treatments were administered daily in a multi-place chamber for 60 min at a depth of 1.75 atmosphere absolute (ATA). After the first ten HBOT treatments, one 25 mg PSE injection was administered approximately once weekly and the number of further HBOT sessions and PSE injections was tailored to the patient. The method of perispinal administration of etanercept was used under license from the patent holder, TACT IP, LLC^1^.

### SPECT Imaging and Visual Analysis

Brain SPECT was carried out before (baseline) and after treatment for each patient. A triple head gamma SPECT camera (Picker Prism 3000XP), equipped with low-energy, ultra-high resolution (LEUHR) fan beam collimators was used to detect the uptake levels of the radiotracer, 99mTc-D, L-hexamethylene-propylene amine oxime (HMPAO), which is correlated with rCBF and metabolic activity. Reconstructions of multiparametric display were performed on the Picker Odyssey computer using filtered back projection and Chang attenuation correction (54). Once the final distribution is established post-injection and without significant change for 2–3 h, the visualization of the whole gray matter volume can be completed *via* a 3-D mapping of perfusion levels. Visual analysis was performed by an expert nuclear medicine physician with over 30 years of experience in SPECT.

In the absence of a qualified biomarker (27, 55), this study evaluates SPECT as an imaging biomarker based on the US Food and Drug Administration (FDA) monitoring biomarker definition (56). Monitoring biomarkers are analyzed at different time points to monitor the status of a disease or medical condition, and as a marker of the response to an intervention (56). In this review, the monitoring biomarker corresponds to the increase in brain perfusion detected with optimized displays of SPECT images before and after treatment. For each patient, the baseline SPECT images were compared with the post-treatment SPECT images to assess the functional improvements across different areas of the brain.

The increased perfusion was detected using a purposefully designed, discrete color scale (DGP40%) as a semi-quantitative tool that assessed relative perfusion across different displays. The distribution of the radiotracer within the brain was visualized in several ways during this study: Firstly, slicing, whereby processing was based on reconstruction, filtering, reorientation and attenuation correction and led to three orthogonal cuts (sagittal, coronal and transaxial) supplemented by a fourth axial display obtained along the temporal axis. Secondly, 3-D stereotactic surface projections were obtained with the Neurostat software (57). Stereotactic surface projection is a technique used for the analysis of SPECT images to extract functional areas projected onto the brain surface for the visual representation of brain perfusion. The discrete DGP40% color scale was applied to the orthogonal slice displays and the surface projection images to facilitate visualization of the level of perfusion. The maximum perfusion in the image was scaled to 100%, with each color band corresponding to a different level of perfusion, as measured in steps of approximately 3%. The threshold is set at 40% to suppress background noise.

Finally, thresholded volumetric displays were used to create a surface that represents voxels of a constant value and are therefore also termed iso-surface images. The region of the brain with the highest uptake of radiotracer was used as the reference value (the cerebellum in the majority of cases) and a 67% threshold value (relative to the reference value) was applied to generate 3-D iso-surface images with “holes” in the image corresponding to areas of the cortex with lower perfusion. Since the images are continuous, the threshold value of 67% was selected as it accentuated corresponding areas in the color images, focused attention on hypoperfused areas and allowed for a better estimation of extent and severity. A threshold of 67% was applied to the iso-surface images shown herein. In addition to the 67% threshold, which depicts areas of hypoperfusion, thresholds of 85 and 90% were also used to create iso-surface images that visualized the size and location of areas of hyperperfusion. The application of multiple thresholds allowed the visualization of hypo- and hyper-perfusion areas across the brain. The clarity, complementarity and user-friendliness of these displays enabled a reliable visual evaluation before and after treatment.

## Results

### Patient I: CTK Treatment

A 62-year-old female presented at the clinic on the verge of suicide. The patient had worked as a nurse prior and following episodes of alcohol abuse. The patient had a history of multiple medication trials and polypharmacy, physical pain and prolonged family stressors (sickness and eventual death of husband) and intense grief. The patient was classified as treatment-non-responsive following multiple treatment failures, which were intended to address her suffering. The patient's formal diagnoses were treatment-resistant depression (TRD) as well as grief and the effects of prolonged polypharmacy.

**Figure 1:**
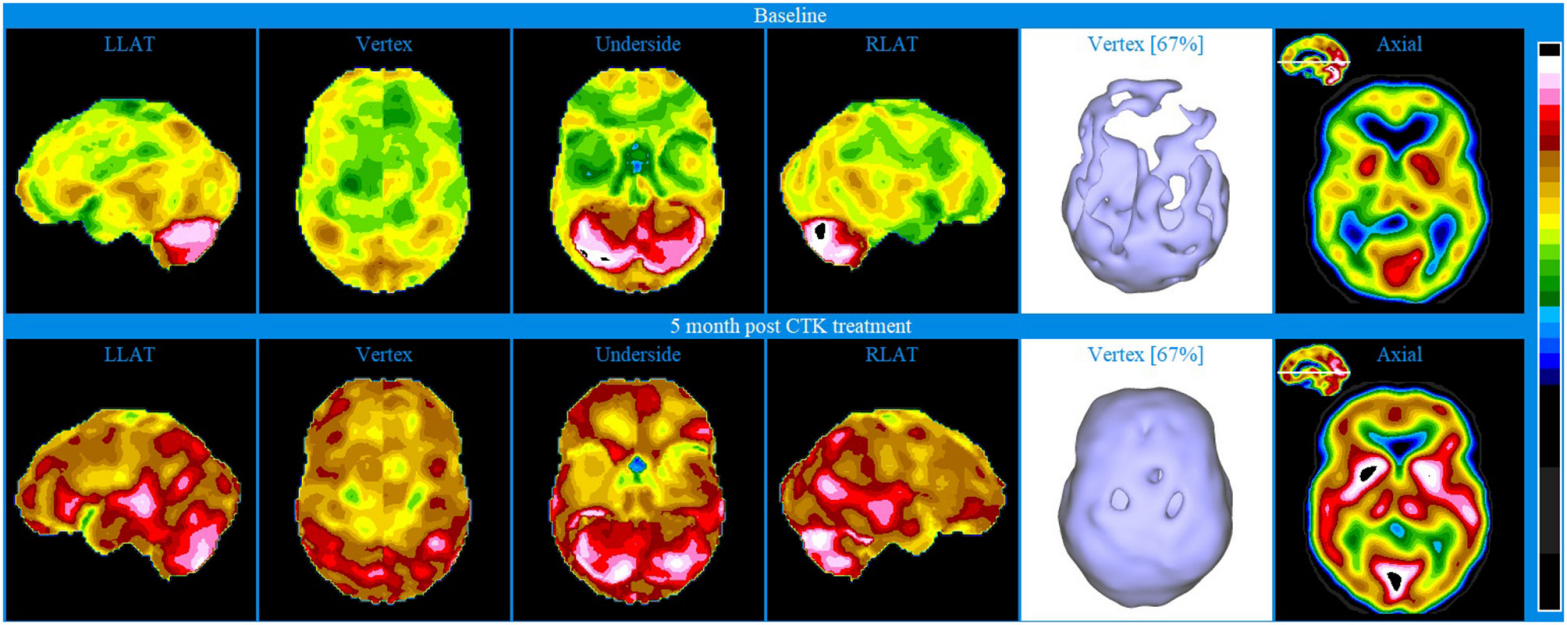
Brain SPECT images from Patient I. Top line images relate to brain SPECT images at baseline. Bottom line images are taken following 5 months of CTK treatment. The six images on each row (from left to right) include four stereotactic surface projections of the left lateral (LLAT), vertex, underside and right lateral (RLAT), one iso-surface image of the vertex and one axial slice. Color-coded intensity indicates hyperfunctioning areas (blue hues) and hypofunctioning areas (white and black surrounded by white). Images are presented as described in the Methods.

Baseline brain SPECT images for Patient I, shown in the top line of Figure 1, indicated a marked and very extensive bilateral hypoperfusion involving all lobes at baseline, and most accentuated on the right side. The more extensive hypoperfusion were located in the lateral frontal, frontoparietal and superior parietal areas. There was marked hypoperfusion in the dorsal aspect of the anterior cingulate. In the subcortical area, there was slight bilateral striatum hyperperfusion and robust perfusion of the thalamus. The patient was then treated with a total of 30 CTK sessions. SPECT images were subsequently taken following 5 months of CTK treatment (58 sessions) and are displayed in the bottom line of Figure 1. These images indicated a markedly improved perfusion across all cortical and subcortical structures. These improvements corresponded with dramatic clinical improvement leading to major changes in her daily life: enthusiastic, rational, planning for future, taking charge of her financial and family situation and a renewed religious sentiment.

### Patient II: HBOT and PSE Treatments

A 34-year-old female presented with lifelong symptoms of regulatory disorder of childhood, two concussions, post-head injury epilepsy, and reflex sympathetic dystrophy (RSD). These ultimately led to marked suffering and extreme disability in activities of daily living. For almost 2 years the patient spent each day in a basement with dark glasses and protective hearing equipment due to intense photophobia and misophonia. Prior to visiting our clinic, the patient's medication history included over 30 types.

**Figure 2:**
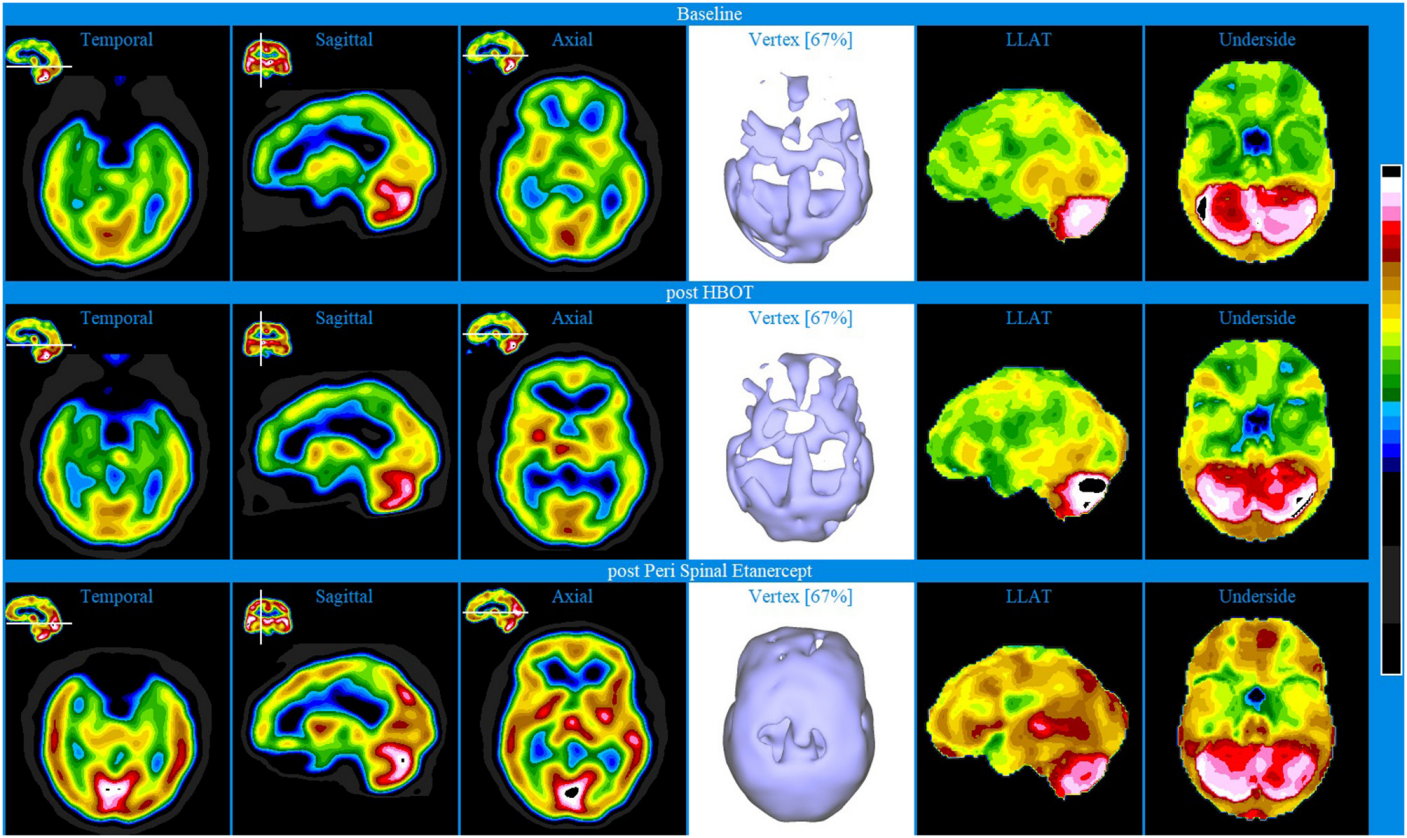
Brain SPECT images for Patient II. Top line relates to brain SPECT images at baseline, indicating extensive areas of hypofunctioning in all lobes. Middle line shows SPECT images post-HBOT treatment. Bottom line of SPECT images collected post-PSE treatment. In each row (from left to right) one temporal axial image, two orthogonal slices (sagittal and axial), one iso-surface image of the vertex and two stereotactic surface projections of the left lateral (LLAT) and underside.

Baseline brain SPECT images for Patient II, shown in the top line of Figure 2, indicated extensive, diffuse bilateral hypoperfusion of the frontal (more accentuated on the left), temporal and orbitofrontal lobes and extended into the frontoparietal and parietal vertex areas. Additionally, there was bilateral hypoperfusion of the occipital lobes and hypoperfusion of the anterior cingulate in the dorsal aspect. The patient had robust perfusion of the thalamus and basal ganglia. There was also marked hyperperfusion in the cerebellar vermis.

HBOT was selected for patient II on account of multiple head injuries and a developmental history of a regulatory disorder. Following 40 HBOT treatments, brain SPECT images (Figure 2 middle line) indicated increased perfusion in most areas of the cortex and in some subcortical structures.

At a later stage, PSE injections were started as another line of intervention. PSE clinical injections were given at weekly intervals. Following 4 PSE injections, SPECT was performed and images shown in the bottom line of Figure 2 indicated major improvements (increased perfusion) in all lobes and subcortical areas. Specifically, the images showed increased perfusion in the orbito-frontal and apico-mesial temporal areas, bilaterally, and in the putamen bilaterally and in the mid thalamus. In addition, there was marked hyperperfusion in the mid posterior/inferior occipital area and, several areas of moderate cortical hyperperfusion in the lateral posterior aspect of both temporal lobes, as well as in the posterior cingulate/precuneus area. Significantly, these improvements were mirrored in the patient's cognition and ability to engage in daily acts of living.

### Patient III: CTK Treatment

A 54-year-old male emergency-room (ER) nurse presented with childhood-onset Tourettes and a long history of alcohol abuse, severe depression, fatigue and sleep apnea.

**Figure 3:**
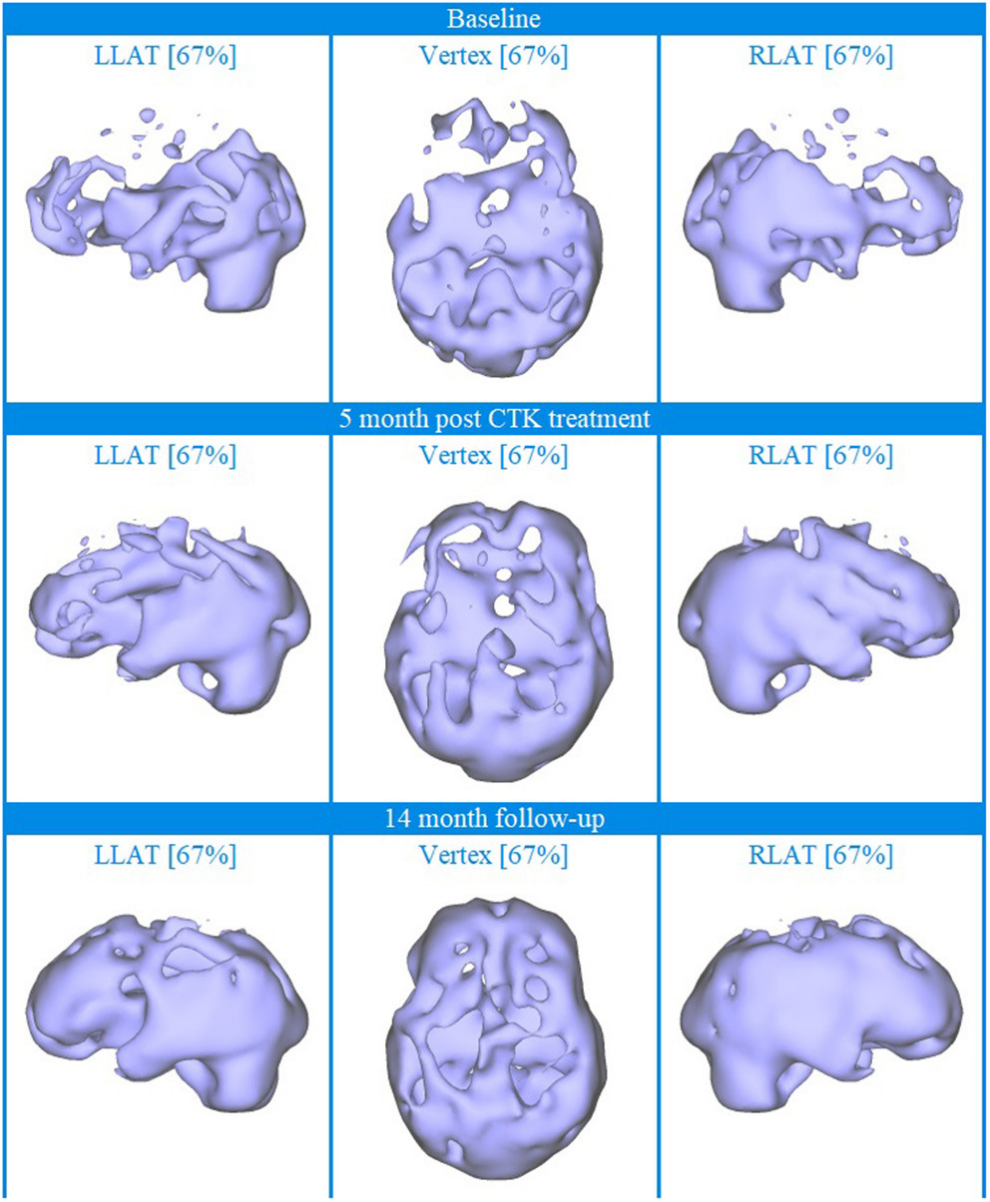
Brain SPECT iso-surface images for Patient III. Top line relates to brain SPECT images at baseline. Middle line shows SPECT images 5 months after the first CTK treatment. Bottom line shows SPECT images 14 months following CTK treatment. In each row (from left to right) three iso-surface images of the left lateral (LLAT), vertex and right lateral (RLAT).

**Figure 4:**
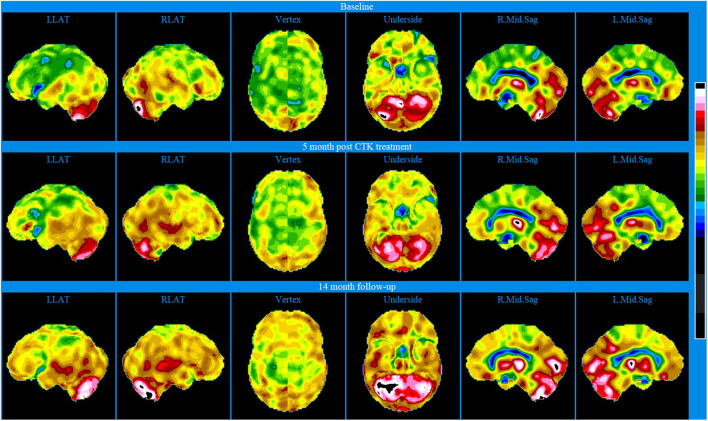
Brain SPECT stereotactic surface projections for Patient III. Top line relates to brain SPECT images at baseline. Middle line shows SPECT images 5 months after the first CTK treatment. Bottom line shows SPECT images 14 months following CTK treatment. In each row (from left to right) six stereotactic surface projections of LLAT, RLAT, Vertex, Underside, right mod sagittal, and left mid sagittal.

Baseline brain SPECT images for Patient III, shown in the top line of Figures 3 and 4, indicated extensive bilateral hypoperfusion with this most accentuated in the left frontal lobe. Additionally, there was localized hypoperfusion in the occipital and frontal poles. On the right side, there were multiple localized and confluent areas of hypoperfusion that were more accentuated in part of the frontal lobe extending to the superior aspect of the parietal lobe including the vertex. Marked hypoperfusion was also evidenced in the right striatum and right ventral striatum, and the thalamus had an asymmetric appearance with localized areas of marked hypoperfusion in the posterior aspect. There was moderate hyperperfusion in the anterior and posterior cingulate.

CTK treatment was selected and SPECT imaging was completed 5 months after the first CTK treatment (middle line of Figures 3 and 4). SPECT indicated several improvements, including in the area of severe frontal hypoperfusion at baseline. Subsequently the patient continued with medication, accepted continuous positive airway pressure (CPAP) treatment, and changed his lifestyle. Nonetheless, he was still unable to change his lifestyle completely at this stage. SPECT imaging completed 14 months later, as shown in the bottom line of Figures 3 and 4, indicated further significant improvements. There were extensive areas of relative increase in blood flow in the lateral aspect of the left hemisphere and bilateral vertex area, along with significantly improved perfusion in the orbitofrontal and apico-mesial temporal areas. Additionally, there was increased perfusion in the left striatum and in the cerebellum. These apparently minor improvements correlated with significant clinical improvements with the patient having resumed working (part-time job), had significantly changed lifestyle with a stable marriage.

### Patient IV: CTK Treatment

A 55-year-old female presented with major depressive disorder (MDD), panic/agoraphobia, chronic back pain and frequent headaches. The neurological exam did not indicate focal neurological dysfunction of the central nervous system. Intermittently, she had been treated with varied pharmacologic interventions and psychotherapy for 24 years, before presenting to this clinic. During that time, the patient's symptoms did not respond to Wellbutrin, Lexapro, Abilify, Viibryd, Paxil, Nardil, Vicodin, nor conventional psychotherapy.

**Figure 5:**
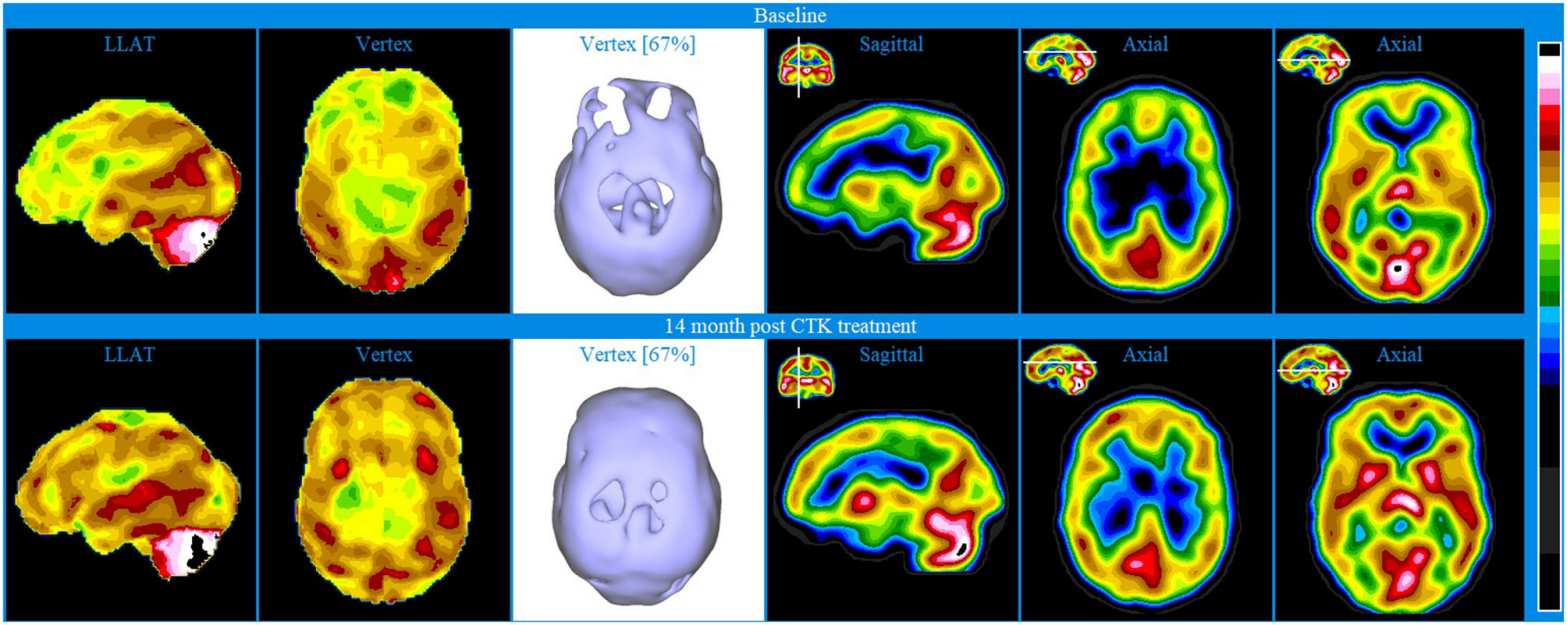
Brain SPECT images for Patient IV. Top line relates to brain SPECT images at baseline. Bottom line shows SPECT images 14 months following 30 sessions of CTK treatment. In each row (from left to right) two stereotactic surface projections of the left lateral (LLAT) and vertex, one iso-surface image of the vertex, one sagittal slice and two axial slices.

Baseline brain SPECT images of Patient IV, shown in the top line of Figure 5, indicated extensive bilateral hypoperfusion in the frontal lobes (most accentuated on the left side), including the dorsolateral prefrontal regions and in the parietal vertex bilaterally. Not discernible in the images provided is the hypoperfusion in both orbitofrontal areas and in the mesial aspect of the right temporal lobe. There was moderate hyperperfusion of the thalamus and marked hyperperfusion in the mid posterior, inferior aspect of the occipital lobes.

The patient was treated with 30 CTK sessions and SPECT images shown in the bottom line of Figure 5 were taken 14 months thereafter. Patient IV also received small amounts of anti-panic medicine that was used judiciously. The SPECT images indicated major bilateral perfusion improvement in the frontal conexities, fronto-parietal and anterior cingulate areas as well as bilateral increase in the basal ganglia. Not discernible in the images provided is the significant improvement of the orbitofrontal areas. There was also significantly increased perfusion in the thalamus and bilaterally in the striatum. Following treatment, the patient reported markedly improved symptoms: major decrease of depression, anxiety and back pain and greatly increased levels of life satisfaction. At the two-year follow-up, the patient had been practically free of suffering.

### Patient V: HBOT and PSE Treatment

A 77-year-old male presented with onset of dementia induced by general anesthesia with major cognitive, physical and emotional impairments. Immediately post knee-replacement surgery the patient began to show dramatic cognitive, physical, and emotional impairment as compared with his pre-surgical state; these symptoms were still present when the patient arrived at our clinic 4 years post-surgery. Diagnoses of dementia with major cognitive deficits and aphasia was established.

**Figure 6:**
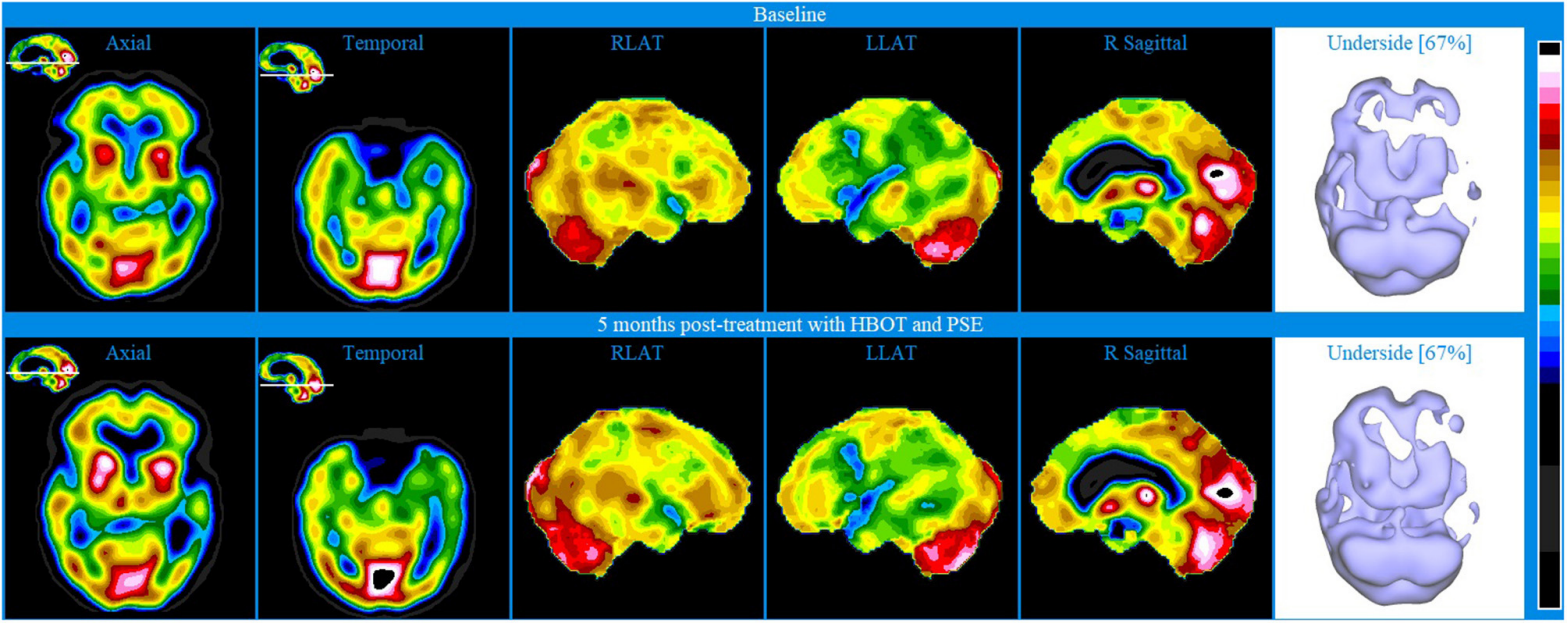
Brain SPECT images for Patient V. Top line relates to brain SPECT images at baseline. Bottom line shows SPECT images 5 months post-treatment with HBOT and PSE. Green arrows indicate areas of increased perfusion. In each row (from left to right) one axial slice and one temporal slice, three stereotactic surface projections of the right lateral (RLAT), left lateral (LLAT) and right sagittal and one iso-surface image of the underside.

Baseline brain SPECT images of Patient V, shown in the top line of Figure 6, indicated extensive hemispheric hypoperfusion and multiple localized hypoperfusion in the left hemisphere. Involvement extended to parts of the dorsolateral prefrontal (DLPF) cortex. There was also significant hypo-perfusion in the temporal lobes (more pronounced on the left) and to a lesser extent in the orbitofrontal areas. To address these cognitive, physical, and emotional impairments, a treatment plan was prepared including a 40-session course of HBOT and PSE injections. After the first 10 HBOT treatments, the patient was administered 25 mg PSE injections approximately once weekly for 5 months.

At 5 months post-treatment with HBOT and PSE, SPECT images shown in the bottom line of Figure 6 showed an overall similar appearance to baseline. However, there were localized increases in perfusion, as marked by the green arrows, in parts of the anterior aspect of the prefrontal cortex (including in the ventro-mesial aspect), right superior parietal, right lateral occipital, superior aspect of the left fronto-parietal area, posterior cingulate-precuneus and apico-mesial aspect of the right temporal. In addition, there was a significant increase in the striatum bilaterally.

Despite the follow-up SPECT remaining abnormal, the improved perfusion in small areas, specifically the mesial temporal lobe, prefrontal cortex, ventro-mesial frontal, posterior cingulate, precuneus and dorsal parietal are known to be key in contributing to memory, cognition and behavior. Indeed, initiating after the first PSE injection, the patient began showing progressive clinical improvements in cognitive and physical function. A follow-up visit 16 months after the end of treatment showed that the same level of clinical improvement had been maintained.

### Patient VI: CTK Treatment

A 43-year-old male presented with bipolar II, lifelong symptoms of depression, anxiety, impulsive behavior and family stressors. Specifically, the patient reported struggling with intense depressed mood, substantial life stress, including a divorce in progress, and the inability to hold a job due to the impairment and distress associated with his symptoms. He had received psychopharmacological and psychotherapeutic treatment for the previous 6 years, but without improvement.

**Figure 7:**
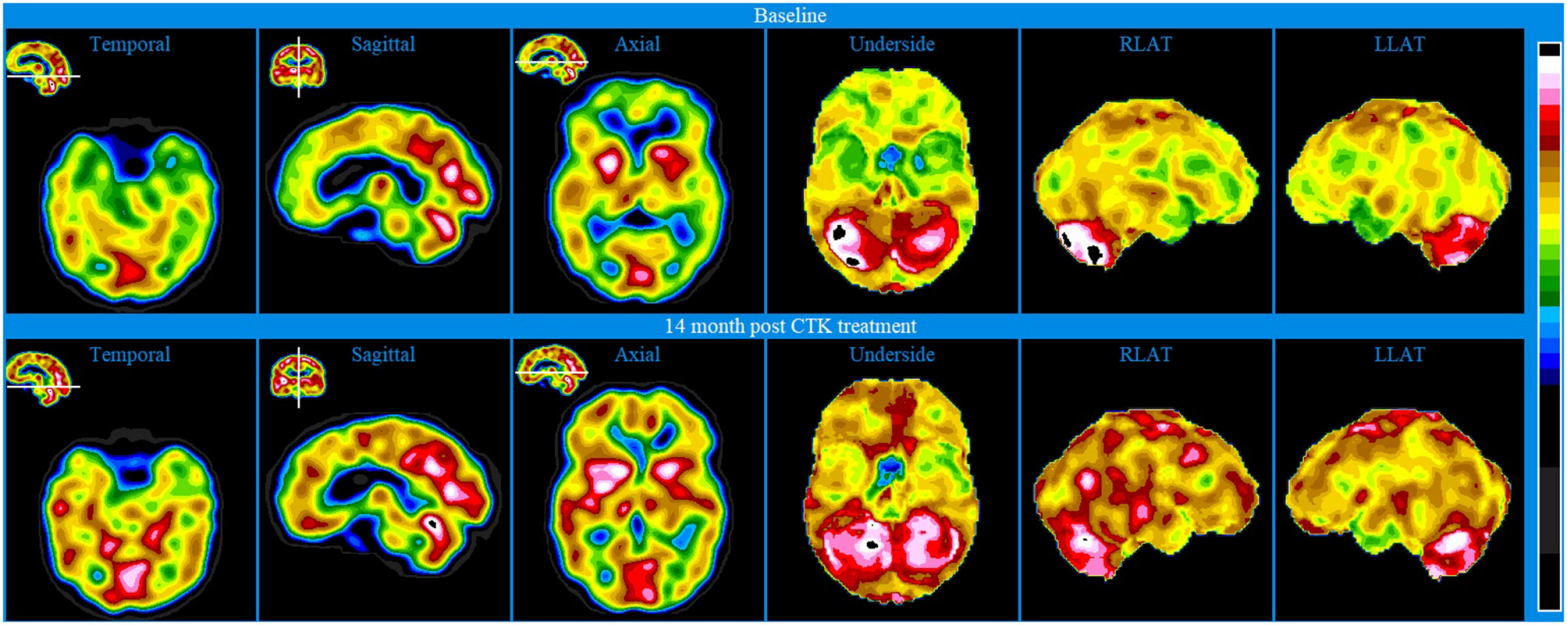
Brain SPECT images for Patient VI. Top line relates to brain SPECT images at baseline. Bottom line shows SPECT images 5.5 months following 24 sessions of CTK treatment. In each row (from left to right) one temporal slice, one sagittal slice and one axial slice and three stereotactic surface projections of the underside, right lateral (RLAT) and left lateral (LLAT).

Baseline brain SPECT images of Patient VI, shown in the top line of Figure 7, indicated hypoperfusion in multiple hemispheric areas, most pronounced in the frontal lobes, anterior cingulate, orbitofrontal and apico-mesial areas of the temporal lobes. Hyperperfusion was indicated in the right putamen and in parts of the posterior cingulate and right cerebellum and vermis. This combination of hypoperfused areas is commonly associated with dysfunctions related to memory, executive function, social interaction and impulse control and the hyperperfused areas are often associated with anxiety and depression (58, 59). Based on the initial assessment, CTK treatment was selected, and the patient received a total of 24 sessions over 5 months.

SPECT was performed 5½ months after the first CTK treatment and images are displayed in the bottom line of Figure 6. Images indicated significantly improved relative perfusion in almost all previously under-perfused areas. Previously hyperperfused areas were either unchanged or increasingly hyperperfused. The patient also reported substantial improvements in symptoms related to functioning and psychometric assessments showed substantial decreases in symptoms related to both depression and mania.

**Figure 8:**
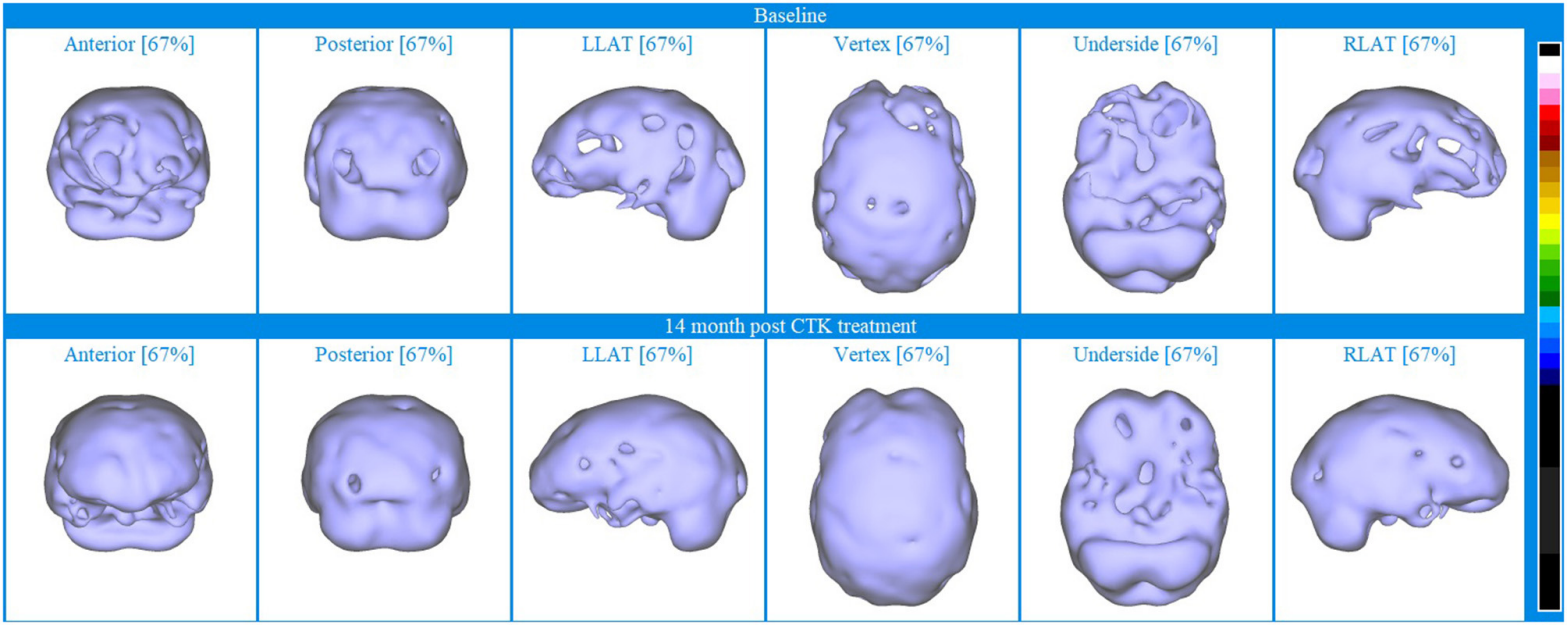
Iso-surface SPECT images for Patient VI. Top line relates to brain SPECT images at baseline and bottom line are brain SPECT images 14 months after CTK treatment began. In each row (from left to right) are iso-surface images of the anterior, posterior left lateral (LLAT), vertex, underside and right lateral (RLAT).

To further display the utility of SPECT as an evaluative biomarker, Figure 8 displays iso-surface images clearly indicating the increased perfusion following CTK treatment in almost all previously hypoperfused areas.

## Discussion

This article describes a novel display technique for SPECT images and provides readers with six case study examples that demonstrate how brain SPECT imaging can be used, both as a complementary diagnostic tool, and as a potential biomarker for monitoring and evaluating novel treatments for patients with complex neuropsychiatric conditions. Developing and applying clinical tools, such as SPECT imaging, can provide the clinician with complementary insights into the underlying neurobiology to standard clinical assessments. However, as SPECT imaging continues to be underutilized in clinical practice, more practical evidence, guidance and studies such as this one are required to demonstrate how SPECT can be utilized to evaluate the patient and to inform the clinician.

In this retrospective review, six patients presented with complex neuropsychiatric conditions and comorbidities following extensive unsuccessful periods of different treatments. While experienced psychiatrists may be able to diagnose patients with neuropsychiatric conditions based on behavioral criteria, functional brain imaging tools can inform the clinician of their underlying neurobiology. Additionally, the examination of different functional areas of the brain using SPECT imaging may provide insights into the patients' status that may not identifiable during clinical assessment. Particularly, in cases of diagnostic dilemma, such as those presented herein, SPECT images were used to identify areas of hypo- and hyper-perfusion to complement the initial clinical assessment. In the cases presented herein, the improved perfusion identified in the post-treatment SPECT images, whether extensive or localized, were mirrored by particular clinical improvements. Therefore, it is fundamental for the clinician to understand how clinical improvements manifest in the patients' underlying neurobiology. This case series effectively demonstrates the importance for the clinician to combine clinical assessments evaluating presenting behaviors and symptoms, with the underlying neurobiology attainable using SPECT imaging. Furthermore, building a database that correlates SPECT images with clinical assessments has the potential to develop more targeted treatments and to establish effective biomarkers.

In these complex cases, high-quality SPECT images provided valuable insights into the underlying neurobiological status of patients allowing the clinician to narrow the differential diagnosis. A high-quality brain SPECT will provide detailed information about the location, magnitude, and extent of areas of hyper- and/or hypoperfusion(s). The clinical efficacy of brain SPECT for the monitoring of patients with multiple co-morbidities and treatment-resistant conditions, is greatly enhanced by a standardized, comprehensive display of the results. This includes a user-friendly, color, multi-parametric set of 2D and 3D images. Consistency is also critically important, not only in the execution of the procedure itself but also in the processing and display of the images. Whilst quantification of SPECT images may facilitate readers with limited experience to interpret SPECT scans and aid in the identification of trends (60, 61), it is imperative to also recognize any limitations of such quantitative analysis of imaging techniques, particularly within clinical practice and during the assessment of individual cases (8). The visual assessment of SPECT images pertaining to individual cases remains a foundational skill within clinical practice, the accuracy and reliability of which relies on the optimization of the presentation of images using effective display tools and techniques. In the clinical cases presented, we have demonstrated that the optimization of images using a novel set of display tools facilitated the visual interpretation of SPECT imaging data by expert clinicians, without the need for quantification or statistical analysis. A more detailed description of this display will be published shortly (62).

In the absence of a qualified biomarker (27, 55), this study evaluates SPECT as an imaging biomarker based on the US Food and Drug Administration (FDA) monitoring biomarker definition (56). A monitoring biomarker is defined as “a biomarker measured repeatedly for assessing status of a disease or medical condition or for evidence of exposure to (or effect of) a medical product or an environmental agent” (56). In this review, the monitoring biomarker corresponds to the increase in brain perfusion detected with optimized displays of SPECT images before and after treatment. Following treatment, all patients demonstrated improvements measured *via* periodic clinical evaluations and in some cases neuropsychological testing and detailed observations from family members. These clinical improvements were also apparent from the increased perfusion evident in the brain SPECT images collected post-treatment compared with those collected at baseline. Whilst clinical improvements following treatment may be discernible during standard clinical assessment, SPECT was used as a monitoring biomarker to indicate the underlying neurobiological response of each individual to the treatment intervention. In this case series, SPECT images indicated the specific areas of improved brain perfusion that resulted following treatment and provided additional context to the clinical improvements identified. Therefore, the SPECT images provided insights into the functioning status of the patient in addition to monitoring symptoms during clinical assessment. These follow-up scans thereby informed how these novel treatments affected the underlying neurobiology of each individual and how perfusion improvements in specific areas correlated to clinical improvements.

The application of SPECT imaging as a monitoring biomarker also further contributes to the understanding and the growing literature that describes the clinical utility of these novel treatments for patients with neuropsychiatric conditions. In this case series, intervention with novel treatments of CTK or HBOT and PSE resulted in marked improvements in relative perfusion in previously hypoperfused areas, which correlated to the clinical improvements noted for each patient. These cases thereby provide further evidence of the clinical utility of these novel treatments for patients with complex neuropsychiatric conditions.

Despite the growing, evidence-based foundation for the application of SPECT in numerous indications relevant to psychiatric practice, there is a need for clinicians to utilize this powerful tool and contribute to our understanding of the neurobiology relating to different neuropsychiatric conditions and comorbidities. Greater biological understanding will result in the identification of meaningful biomarkers for diagnosis, prognosis or risk and can further aid the clinician in their evaluation of a patient before and after treatments. There is a particular value in sharing methodologies and results regarding the implementation of SPECT in routine practice in clinical settings. This retrospective review demonstrated that brain SPECT imaging could represent a potential imaging biomarker since syndrome status was correlated with changes in the perfusion pattern detected. Given the display modalities used, the relative perfusion assessment with SPECT imaging before and after treatment has acted as a monitoring biomarker that indicated the therapeutic benefit of the novel types of treatments used in this case-series. Furthermore, SPECT images have provided additional information that explains the functional changes that gave rise to the observed clinical improvements. This understanding of the topographic functional status is important if we are to further progress to personalized targeted treatments and the development of effective biomarkers.

This article has some limitations. First, these cases represent assessments carried out during routine clinical practice and not as part of a pre-planned study, therefore there is a small number of cases presented without a normalized reference cohort and physicians were not blinded to the clinical context, however this does reflect a practical clinical routine.

Overall, this collection of these case studies further substantiates the clinical relevance of brain SPECT imaging in psychiatry and neuropsychiatry. This review demonstrates that SPECT imaging can be a valuable tool in cases of diagnostic dilemma and can complement standard assessment techniques and diagnostic tools. In our study, six patients with complex neuropsychiatric conditions and comorbidities were successfully treated, which contributes to the growing literature indicating the clinical utility of the novel therapies of CTK and HBOT with PSE (33, 47). The positive outcomes for these patients were facilitated by the detailed initial evaluation of patients that included baseline SPECT imaging that complemented the standard clinical assessment. The repetitious clinical approach, the novel display technique and positive treatment outcomes in these six cases has also demonstrated how image optimization and visual analysis of SPECT images can be utilized during clinical assessments of individual cases. Finally, we demonstrated how SPECT images recorded before and after treatment provided valuable insights into the improved neurobiological status of these patients in response to intervention. Therefore, we argue that perfusion assessed with SPECT images before and after treatment can be used as an imaging biomarker for monitoring, evaluating and explaining clinical change.

## Author's Note

Proper execution of a brain SPECT scan is an art and requires great attention to detail in order to obtain high quality images, such as the ones presented here. For specific information regarding imaging and processing protocols, please contact the authors directly.

## Data Availability Statement

The datasets presented in this article are not readily available because HIPAA-protected data. Requests to access the datasets should be directed to srdbest@neuroscience.md.

## Ethics Statement

Ethical review and approval was not required for the study on human participants in accordance with the local legislation and institutional requirements. The patients/participants provided their written informed consent to participate in this study. Written informed consent was obtained from the individual(s) for the publication of any potentially identifiable images or data included in this article.

## Author Contributions

SB provided the CTK, HBOT and PSE treatments, as well as integrated the psychiatric and SPECT data. NH co-wrote the article and contributed to the discussion of the results. DP performed the SPECT imaging, as well as analyzed and interpreted the SPECT data. Please note that DP sadly passed away during the finalization of this manuscript, however all other authors read and approved the final manuscript.

## Conflict of Interest

NH has since become an employee of Eli Lilly and Company. SB holds multiple patents claiming methods of use of CTK for treatment of neurological disorders. The remaining author declares that the research was conducted in the absence of any commercial or financial relationships that could be construed as a potential conflict of interest. The handling editor TH declared a past co-authorship/collaboration with one of the authors DP.

## Publisher's Note

All claims expressed in this article are solely those of the authors and do not necessarily represent those of their affiliated organizations, or those of the publisher, the editors and the reviewers. Any product that may be evaluated in this article, or claim that may be made by its manufacturer, is not guaranteed or endorsed by the publisher.

## Footnotes

^1^The method of perispinal administration of etanercept utilized was used under license from the patent holder, TACT IP, LLC, Boca Raton, FL, USA. TACT IP LLC claim methods of use of etanercept for the treatment of neurological disorders, including, but not limited to, US patents 6419944, 6537549, 6982089, 7214658, 7629311, 8119127, 8236306, and 8349323.
